# Verses

**Published:** 1906-11

**Authors:** 


### VERSES.

---

### A YOUNG LADY OF BOSTON.

There was a young lady of Boston,
Whose manner had such a deep frost on
    She invariably froze
    Every one of her beaux
When her high plane of thought they
        got lost on.

—Life.

### THE GOSPEL OF ART.

Work thou for pleasure, paint or sing or carve
The thing thou lovest though the body starve.
Who works for glory misses oft the goal;
Who works for money coins his very soul.
Work for the work's sake then, and it may be
That this thing shall be added on to thee.

—Kenyon Cox.

### THE DIFFERENCE ON A FROSTY MORN.

De blackman gits up on er frosty morn
An e draws up en er knot,
An e goes out slow ter his daily job,
But e starts er fire rite on de spot.
An e slaps his arms er time or two,
Den e bends ober dat fire,
An e toasts his hans an den his toes
Consarnin not time nor loss ob hire.
De wite man gits up on er frosty morn,
An e hustles eroun an erbout,
An e rubs his ears an stamps his feet,
Den gits out doors wid er shout.
An e tackles his wuck an keeps agoin,
By dis time e's een er swet,
Doan lose no time by foolin wid fire
An e gits his hire yer jess better bet.

—B. H. Teague.

### THE SONG OF THE FAINT.

Take her up tenderly,
Mind her back hair,
Fashioned so slenderly,
Fetch her a chair!
What can the matter be?
What's her complaint?
Send for the doctor, she
'S off in a faint!
What is the mystery?
Tell us the history
How it began,
Tell us for pity's sake—
See! she's now awake!
Give me a fan!
Oh, what an awful moan!
Get me salts and cologne
Quick from the house.
Give her air, keep away!
What does the lady say?
She saw a mouse!

—La Touche Hancock.

